# Outcomes following hip and knee replacement in diabetic versus nondiabetic patients and well versus poorly controlled diabetic patients: a prospective cohort study

**DOI:** 10.1080/17453674.2018.1473327

**Published:** 2018-05-14

**Authors:** Erik Lenguerrand, Andrew D Beswick, Michael R Whitehouse, Vikki Wylde, Ashley W Blom

**Affiliations:** 1 Musculoskeletal Research Unit, Translational Health Sciences, Bristol Medical School, University of Bristol, Bristol;; 2 National Institute for Health Research, Bristol Biomedical Research Centre, University Hospitals Bristol NHS Foundation Trust and University of Bristol, UK

## Abstract

**Background and purpose** — The impact of diabetes and glycemic control before joint replacement on clinical and patient-reported outcomes is unclear. We compared pain, function, complications, and length of hospital stay in diabetic and nondiabetic patients receiving primary total hip (THR) or knee replacement (TKR) and compared these outcomes in patients with poorly controlled versus well-controlled diabetes.

**Patients and methods** — We conducted a prospective cohort study of patients undergoing primary THR (n = 300) or TKR (n = 287) for osteoarthritis. Self-reported diabetes and glycemic control (HbA1c ≤ or >7%) extracted from medical notes were used. Adjusted comparisons were performed with generalized linear models including body mass index (BMI) and comorbidities.

**Results** — Diabetes prevalence was 11% (THR 8%; TKR 14%). Diabetic patients were more likely to have a higher BMI and greater number of comorbidities. The median length of hospital stay was 1 day longer in diabetic patients (p = 0.004), but this attenuated after adjustments for BMI and comorbidities (p = 0.3). Inpatient pain was greater for diabetic patients but attenuated following adjustment. The 12-month postoperative WOMAC subscales were similar by diabetes status following adjustment. There was little evidence of difference in outcomes according to glycemic control.

**Interpretation** — The associations between diabetes and worse postoperative outcomes in patients undergoing THR or TKR for osteoarthritis appear to be predominantly due to associated obesity and comorbidities. In diabetic patients there is little evidence of association between postoperative outcome and preoperative glycemic control. The underlying mechanisms and causal pathways of obesity, diabetes, and multimorbidity that lead to worse outcomes after joint replacement are not well known.

Multimorbidity is common among people undergoing joint replacement, with approximately 70% of patients reporting at least 1 condition additional to osteoarthritis. Prevalent comorbid conditions include degenerative disc disease, osteoporosis, visual and hearing impairment, anxiety (Blom et al. 2016).

In the USA, about 14% of people with osteoarthritis have diabetes (Bhattacharyya et al. 2002) and in a large UK cohort, approximately 6% and 10% of patients undergoing THR and TKR respectively reported having diabetes (Arden et al. 2017). After joint replacement surgery, higher rates of complications such as pneumonia, stroke, bleeding (Bolognesi et al. 2008) and prosthetic joint infection (Kunutsor et al. 2016) are experienced by diabetic patients. Some studies report that diabetes does not affect pain and function after joint replacement as measured by patient-reported outcome measures (Cushnaghan et al. 2009, Clement et al. 2013), but at least 1 study has shown transient worse functional outcome in diabetic patients after TKR, which resolved over time (Robertson et al. 2012).

The importance of tight glycemic control before surgery in diabetic patients is contentious. A USA study of nearly 1 million patients undergoing hip or knee replacement reported that the risk of postoperative complications was greater in the 3.6% of patients with poorly controlled diabetes based on criteria including self-monitoring, treatment and HbA1c > 7% (Marchant et al. 2009). However, a more recent systematic review of cohort studies showed that an HbA1c > 7% before surgery, reflecting poorly controlled diabetes, was not consistently associated with postoperative morbidity or mortality (Rollins et al. 2016).

The aims of this study were 2-fold. First, we compared complications, pain, function, and length of stay in diabetic and nondiabetic patients receiving a primary THR or TKR. Second, we compared these outcomes between patients with poorly controlled diabetes and well-controlled diabetes.  

## Patients and methods

The reporting of this cohort study follows STROBE guidelines.

### Study population

We performed a secondary analysis of data from participants in the Arthroplasty Pain Experience (APEX) trials (Wylde et al. 2015). Between 2009 and 2012, 322 patients receiving primary THR and 316 patients receiving primary TKR in the UK were recruited into 2 single–center randomized controlled trials that investigated the effectiveness of local anesthetic wound infiltration in reducing chronic pain after joint replacement. Patient inclusion criteria were a primary unilateral THR or TKR for osteoarthritis. Exclusion criteria included inability to provide informed consent or complete questionnaires, and medical comorbidity precluding use of spinal anesthesia, regional blocks, and strong analgesics postoperatively.

The study sample included the APEX participants who underwent surgery and reported their diabetic status; patients who withdrew prior to surgery or during the inpatient stay were excluded. Details of the sample size calculation for the original trial have been published previously (Wylde et al. 2015).

### Patient characteristics

Information on age, sex, and BMI were extracted from participants’ medical records by a research nurse. Self-reported comorbidities were collected preoperatively with the 18-item Functional Co-morbidity Index (FCI) (Groll et al. 2005). Psychological distress was assessed with the Hospital Anxiety and Depression Scale (Zigmond and Snaith 1983). Health-related quality was measured using the EQ-5D (Williams and Kind 1992). Participants completed the 24-item Western Ontario and McMaster Universities Osteoarthritis Index (WOMAC) (Bellamy et al. 1988). This tool has 3 dimensions: pain, stiffness, and physical function. Separate subscale scores were calculated and transformed to a 0–100 scale (worst to best). Treatment allocation in the original trial was also considered.

### Diabetic status

Preoperative diabetic status was assessed as part of the FCI, which asks patients whether they have been diagnosed with diabetes. The FCI does not differentiate between type 1 and type 2 diabetes. During the preoperative clinic assessment a research nurse screened the medication usage of all patients for diabetes medication. Relevant reported drugs are listed in Table 1 (see Supplementary data). Diabetic patients were classified in two groups depending on their glycemic control as determined by preoperative HbA1c with a cut-off of >7%(53 mmol/mol) (American Diabetes Association 2017).

### Postoperative outcomes

Outcomes collected during the inpatient stay included the length of hospital stay and acute postsurgical pain. Participants were asked to “indicate the intensity of your present hip/knee pain” at rest and on movement for 3 days post-surgery using a visual analogue scale from 0 to 10 cm (best to worst). The mean VAS scores at rest and on movement across the 3 postoperative days were calculated.

At 3 and 12 months post-operation a research nurse extracted details of postoperative complications from the medical notes. These included: infection; dislocation; deep vein thrombosis; pulmonary embolism; nerve damage; hospital readmission related to the index operation; and further surgery on the index joint. The number of inpatient hospital stays, day case, and outpatient visits related to the index joint were also collected at the 12-month follow-up.

Participants were sent a postal questionnaire at 3, 6, and 12 months postoperatively, which included the WOMAC questionnaire. The WOMAC pain subscale measured at 12 months postoperatively was the primary outcome of the APEX trials.

### Statistics

THR and TKR patients were analyzed together to ensure a sufficient sample of diabetic patients with good and poor glycemic control.

Patient characteristics and outcomes are reported by diabetic status and glycemic control (HbA1c level ≤ or >7%) using frequencies and percentages for categorical variables and means (SD) or medians with percentiles (25th, 75th) for continuous variables, depending on their distribution.

**Figure 1.**
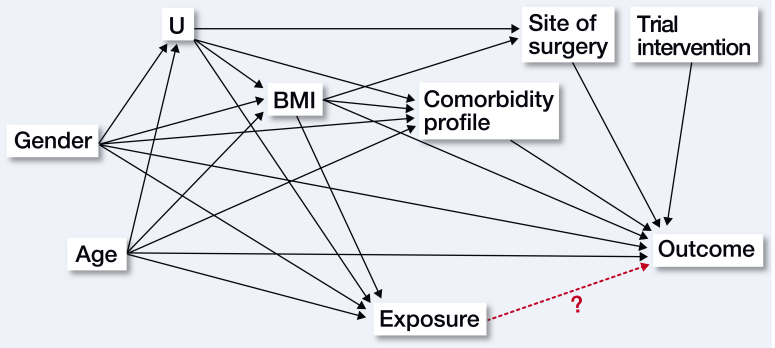
Directed acyclic graph depicting the potential associations underlying the effect of the main exposures on the outcomes. U: unmeasured dietary habits and sedentary lifestyle factors. Exposure: diabetes mellitus or HbA1c status. Outcome: patient reported, clinical or complication outcomes.

We first investigated the unadjusted relationship between diabetes status or glycemic control (exposure factors) and the different outcomes. Multivariable regression models were then fitted to condition these associations on age, sex, site of surgery, BMI, and number of comorbidities and therefore determine whether the exposure factors had an independent and direct relationship with the studied outcomes. These covariates were imbalanced between exposure levels (Tables 2 and 5, see Supplementary data) and have evidence of association with the outcomes in the literature (Judge et al. 2010, 2012) and in univariable models. As shown in the directed acyclic graph (Figure 1), none of them were expected to be colliders but were required in the models to “block open-paths”, i.e., to control for confounding bias or “non-causal structural associations” (Shrier and Platt 2008). The first multivariable models included adjustment for the non-modifiable factors age, sex, site of surgery, and trial intervention. The variable “trial intervention” was forced as it is related to the design of the primary study for which the analyzed data were originally collected. BMI and comorbidity profile—(partially) “modifiable factors” in the perioperative period with non-surgical or pharmaceutical interventions—were finally added to these first multivariable models to identify their specific impact on the exposure–outcome associations.

To account for repeated measures of the studied outcomes, we used linear mixed models for unadjusted and adjusted analyses. The model residuals were not normally distributed (checked with normal quantile–quantile plot) due to outcome distribution heterogeneity across the postoperative period: the scores were normally distributed until 3 months postoperatively but substantially skewed thereafter, in particular at 12 months when numerous patients experienced no or minor symptoms. Each outcome assessment was therefore separately investigated. Unadjusted investigations were performed with unpaired Student t-tests or Mann–Whitney tests for continuous variables and chi-square or Fisher’s exact test for categorical variables. For the adjusted investigations, multivariable linear regression models were used. In the absence of appropriate Box–Cox transformation to use the linear regression model in agreement with its assumptions, outcomes were categorized and analyzed with an ordered logistic regression model. Length of hospital stay was modelled as an ordered categorical variable, ≤ 3, 4, 5, and >5 days (respectively 20%, 29%, 22%, and 29% of the sample). The WOMAC scores were transformed using previously published threshold definitions (severe [0–50], moderate [51–75], mild [76–99], no [100] symptoms) (Wylde et al. 2015). The proportionality assumption was investigated with the Brant test and none of the presented models violated this assumption.

Multiple imputation by chained equations (MICE) was also performed to generate 20 imputations sets and investigate bias induced by missing data on the estimations of the complete-cases analyses (White et al. 2011): patients with high BMI or larger number of comorbidities had more missing item information for the postoperative WOMAC scores. The imputation model included all variables used in the adjusted model, variables presented in Table 1, and all repeated measurements of the studied outcomes. Estimates were combined using Rubin’s rules.

**Table 2.** Sample characteristics by diabetic status at preoperative assessment. Values are number (percentage) unless otherwise stated

	Total sample	Nondiabetic patients	Diabetic patients
	n = 587	n = 523	n = 64
Trial intervention			
Injection	294 (50)	263 (50)	31 (48)
Standard care	293 (50)	260 (50)	33 (52)
Primary total joint replacement			
Hip	300 (51)	276 (53)	24 (37)
Knee	287 (49)	247 (47)	40 (63)
Age, mean (SD)	68 (10)	68 (10)	70 (8)
Sex			
Female	325 (55)	295 (56)	30 (47)
Male	262 (45)	228 (44)	34 (53)
BMI, mean (SD)	31 (6)	31 (6)	34 (6)
≤ 30	303 (52)	282 (54)	21 (33)
> 30	284 (48)	241 (46)	43 (67)
EQ VAS^a^, n	553	492	61
mean (SD)	66 (20)	66 (20)	64 (19)
EQ-5D Score^b^, n	555	495	60
mean (SD)	0.42 (0.32)	0.43 (0.32)	0.37 (0.31)
FCI^c^ (Number of comorbidities)			
	192 (33)	178 (34)	14 (22)
2	168 (29)	156 (30)	12 (19)
3	115 (20)	102 (19)	13 (20)
	112 (19)	87 (17)	25 (39)
HADS^d^ Total, n	567	505	62
mean (SD)	13 (7)	13 (7)	13 (6)
WOMAC^e^ Pain, n	587	523	64
mean (SD)	42 (17)	43 (18)	41 (15)
WOMAC Function, n	558	499	59
mean (SD)	44 (18)	44 (18)	43 (16)
WOMAC Stiffness, n	548	487	61
mean (SD)	44 (22)	43 (22)	44 (18)
WOMAC Total, n	537	479	58
mean (SD)	44 (17)	44 (17)	44 (14)

a EQ visual analogue scale (0–100, worst to best health state).

b EQ-5D 3L descriptive system.

c Modified Functional Co-morbidity Index without BMI and diabetes diagnoses (categorized sum of the 16 remaining diagnoses).

d Hospital Anxiety and Depression Scale with anxiety and depression scores combined (0–42, best to worst distress state).

e Western Ontario and McMaster Universities Osteoarthritis Index (0–100 worst to best pain, function, stiffness or total score).

Analyses were conducted in Stata (Stata Statistical Software: Release 14.1—StataCorp LLC, College Station, TX, USA).

### Ethics, registration, funding, and potential conflicts of interest

The original trials were registered in the Clinical Trial Registry (ISRCTN96095682) and had received ethics approval (NHS-REC South West 09/H0504/94). All participants provided informed written consent. This article presents independent research funded by the NIHR under its Programme Grants for Applied Research program (RP-PG-0407-10070). The authors acknowledge the support of the NIHR, through the Comprehensive Clinical Research Network.

This study was also supported by the NIHR Biomedical Research Centre at University Hospitals Bristol NHS Foundation Trust and the University of Bristol. The views expressed in this publication are those of the authors and not necessarily those of the NHS, the National Institute for Health Research or the Department of Health.There are no conflicts of interest to be reported by the authors.

## Results

### Participants

A total of 304 participants undergoing THR and 290 participants undergoing TKR had complete preoperative information and remained in the APEX trials at the time of their discharge from hospital. Seven patients with no details of their preoperative diabetic status were excluded. Of the remaining 587 patients, 29 (24 nondiabetic and 5 diabetic patients) withdrew from the study or died during the follow-up period (Table 2).

The trial intervention to which patients had been allocated was balanced between diabetic and nondiabetic patients. The mean age of participants was 68 years (SD 10) and 55% were female. The prevalence of diabetes in the overall sample was 11%, higher in the TKR group than in the THR group (14% vs. 8%). Diabetic patients were more likely to have a higher BMI (mean (SD): 34 (6) vs. 31 (6) for nondiabetic patients) and more comorbidities (mean (SD): 5 (2) vs. 3 (2) comorbidities).

All patients with a medication for diabetes had correctly reported their diabetes in the FCI. Of the 64 diabetic patients, 38 were on medication for diabetes prior to their surgery. All treated patients were on metformin tablets; 6 had also been prescribed insulin injection and 1 had been prescribed exenatide injection.

### Postoperative outcomes by diabetic status

A comparison of postoperative outcomes by preoperative diabetic status is provided in Table 3 (see Supplementary data). Diabetic patients had a longer hospital stay than those without diabetes (5 vs. 4 days, p = 0.004). This difference was not explained by age, sex, or the type of surgery, but was not apparent when BMI and the comorbidity profile were accounted for. During the first 3 days of their inpatient stay, diabetic patients reported a higher mean pain level on movement and at rest, but differences were not apparent when adjustments were included for BMI and the comorbidity profile.

No evidence of differences in postoperative complications were observed at either the 3- or 12-month time points. The number of inpatient stays, and day case and outpatient visits related to the operated joint did not differ between diabetic and nondiabetic patients.

WOMAC pain, function, and stiffness at the 3-month and 6-month assessments did not differ between diabetic and nondiabetic patients. At 12 months, diabetic patients reported worse pain, function, and stiffness, but these differences were partially attenuated when the linear regression model included adjustments for age, sex, site of surgery, and trial intervention and not evident with further adjustments for BMI and number of comorbidities. Similar results were found in the imputed analyses (Table 4, see Supplementary data).

### Postoperative outcomes by HbA1c level for patients with diabetes

A comparison of postoperative outcomes by HbA1c level for patients with diabetes is provided in Table 6 (see Supplementary data). 25 of the 64 diabetic patients had a preoperative HbA1c level >7%. Their mean HbA1c was 8.5% (SD 1.6) compared with 6.2% (SD =0.6) for patients with HbA1c ≤ 7% (Table 6, see Supplementary data). Patients with HbA1c > 7% had greater preoperative stiffness and a lower quality of life, but were less likely to have more than 3 comorbidities. They were also more likely to be on medication for their diabetes.

Comparison of outcomes in diabetic patients by HbA1c level revealed little difference between groups. The median length of hospitalization did not differ between HbA1c groups. Inpatient postoperative VAS pain scores were also comparable. Those with a preoperative HbA1c ≤ 7% maintained lower levels of postoperative HbA1c compared with those who had higher preoperative HbA1c level, but no evidence of pre–postoperative change in the level of HbA1c was found within each group (paired t-test: p = 0.7 within the HbA1c ≤ 7% group; p = 0.2 within the HbA1c > 7% group).

After adjustments for confounding factors, diabetic patients with HbA1c >7% reported more pain on the WOMAC scale than those with HbA1c < 7% at 6 months but not 3 months or 12 months postoperatively.

Little evidence of difference in the WOMAC stiffness scores was found between the 2 HbA1c groups at the 3-month and 6-month assessments, but at 12 months postoperatively patients with HbA1c > 7% reported a higher level of stiffness. This difference remained significant in the adjusted comparisons and after adjusting further for preoperative level of WOMAC stiffness (p < 0.0001).

WOMAC function and total WOMAC scores were similar between HbA1c groups.

The median numbers of post-surgery complications, inpatient stay, day case or outpatient visits were also comparable between the HbA1c ≤ 7% and HbA1c > 7% groups. Similar results were found in the imputed analyses (Table 7, see Supplementary data).

## Discussion

In this study of 587 patients receiving THR or TKR, unadjusted analysis revealed that patients with diabetes had longer hospital stays, more severe acute postoperative pain, and worse patient-reported outcomes at 12 months after surgery than nondiabetic patients. However, when the analysis included adjustment for comorbidities and BMI, these differences in outcomes were no longer apparent. This adds to the existing knowledge by showing that diabetes per se may not be related to poorer outcomes after joint replacement, independent of obesity and multimorbidity.

This study has limitations that should be acknowledged when interpreting the results. THR and TKR patients were analyzed together to ensure a sufficient sample in which to investigate disparities in outcome by diabetes and glycemic status. Although the multivariable analyses were adjusted for surgical site, including both TKR and THR patients in the analysis may have confounded the results because of differences between these 2 groups, such as a higher prevalence of diabetes in patients with knee osteoarthritis and poor outcomes after TKR. Investigation of the associations between diabetes/glycemic status and outcomes was limited to patient characteristics collected in the original trial and based on an observational study design approach. Residuals and unmeasured confounding cannot be ruled out and the few independent associations observed in this analysis may be explained by other unobserved factors. This limitation does not affect the associations that were explained by the observed confounders accounted for in the adjusted models. The sample size of this study was initially determined by a power calculation used to address the primary research aim of the original trial. As such, the comparisons by diabetic status performed in this study should be interpreted with care and acknowledgement that a larger sample and/or different study design may be required to draw definitive, causal conclusions. Comparisons by HbA1c status were certainly underpowered and therefore should be considered as purely exploratory. A larger sample, such as that provided by a national arthroplasty registry linked to national primary, secondary, and tertiary care use registries, would be required to investigate the effect of diabetes on rare complications such as infection (Lenguerrand et al. 2017); however glycemic status, pain, or function outcomes are not collected on all patients recorded in these large datasets. Very few studies have investigated the role of diabetes and HbA1c on both clinical complications and longitudinal patient-reported outcomes, especially outside the USA. Our analysis adds to the existing literature, and highlights the need for further research, particularly in a European context.

Diabetic status was identified using the patient-reported FCI tool. This self-reported status was cross-checked with the preoperative list of medications and all patients with a medication for diabetes had correctly reported their diabetes in the FCI. Although all patients underwent routine preoperative blood glucose measurement, it is possible that some patients in our comparator group may have had undiagnosed and untreated diabetes. Type 1 and 2 diabetes cannot be differentiated in the FCI and were thus analyzed together. Type 1 diabetes has a greater negative impact on postoperative complications than Type 2 diabetes, and therefore it may have been more appropriate to have excluded or analyzed these patients separately (Viens et al. 2012). However, most treated patients in this study were on metformin tablets only, suggesting that this lack of differentiation by diabetic type is likely to have little impact on our results, which are more generalizable to those patients undergoing lower joint arthroplasty with Type 2 diabetes, the most frequent type among adults. No information on the duration and severity of the disease were available. Also, no information was available on the number of diabetic patients who had their surgery delayed until their glycemia was controlled/treated, nor about the length of any delay. At the time of the study it was not unit local policy to delay surgery for poor glycemic control. Finally, this work is an ad-hoc, un-prespecified secondary analysis of data initially collected for a randomized trial. The selection criteria (Wylde et al. 2015) used to identify and recruit patients into the original trial were not related to diabetes and are not likely to have introduced any selection bias towards a particular group of diabetic/nondiabetic patients. The patients randomized had also been found to be representative of patients receiving hip and knee replacement in England and Wales. Moreover, the multivariable analyses included an adjustment for intervention allocation. The risks of selection bias associated with the secondary use of randomized trial data and “intervention” bias are low in this analysis.

Extended postoperative hospitalization for patients with diabetes has been reported previously in orthopedic populations (Marchant et al. 2009, Lovecchio et al. 2014, Winemaker et al. 2015), and diabetic patients are more likely to have a non-routine discharge (Bolognesi et al. 2008, Marchant et al. 2009). However, most of these studies did not adjust for BMI and key comorbidities, 2 sets of factors strongly associated with diabetes. Our results imply that extended hospitalization is driven by clinical characteristics commonly encountered in diabetic patients rather than by diabetes in isolation. Indeed, within our sample of patients without diabetes, multimorbidity and obesity were also associated with prolonged hospitalization. Efforts to decrease length of stay should thus target those with obesity and multimorbidity, irrespective of the presence of diabetes.

In terms of complications, our analysis found that rates of postoperative complications, hospital readmissions, and day-case or outpatient visits were comparable between patients with and without diabetes. This may be due to the low rate of postoperative complications after joint replacement meaning that our study was underpowered to detect differences despite having nearly 600 patients.

Previous research that has evaluated the relationship between diabetic status and functional outcomes after joint replacement has reported conflicting results (Gandhi et al. 2010, Singh and Lewallen 2013). We found that patients with diabetes had worse functional results, but not when obesity and multimorbidity were accounted for in the models.

In patients with diabetes, functional outcomes and length of stay were comparable between those with good and poor glycemic control. Some large observational studies have found a detrimental effect of uncontrolled glycemic level on these outcomes (Marchant et al. 2009, Jamsen et al. 2010), but our findings are in agreement with a systematic review published on this topic, which concluded that elevated preoperative HbA1c was not definitively associated with increased postoperative morbidity or mortality in patients with diabetes (Rollins et al. 2016). No, or weak, evidence of an association between diabetes, HbA1c level, and all-cause rehospitalization has previously been reported (Adams et al. 2013). Concerns have been raised as to suboptimal management of diabetes in surgical patients in the UK, particularly with reference to optimization of blood glucose control (Howieson et al. 2014). Our work suggests that in the joint replacement setting optimal preoperative glycemic control may not be critical in determining outcome. This gives further credence to our finding that diabetes is not the major driver of extended hospitalization in diabetic patients undergoing joint replacement. Our findings regarding pain are mixed, with evidence of increased pain in those with worse glycemic control at earlier time points, but no differences in the longer term.

In our analysis that combined patients undergoing THR and TKR, we found no association between the presence of diabetes and postoperative stiffness. In contrast other authors have reported increased stiffness in diabetic patients undergoing TKR (Bawa et al. 2013); evidence for THR is scarce. As we included patients undergoing THR this may explain the difference with those studies. However, we did find that patients with poorly controlled diabetes (72% of them had undergone TKR) had a higher level of stiffness than those with well-controlled diabetes. This may be due to increased scar formation in hyperglycemic patients or difficulty for those with poorly controlled diabetes in complying with their postoperative rehabilitation leading to stiffness.

In summary, we found that the associations between diabetes and worse postoperative outcomes were due to obesity and comorbidities. In diabetic patients there is little evidence of associations between preoperative glycemic control and postoperative outcomes. Further research is required to understand the underlying mechanisms and causal pathways of obesity, diabetes, and multimorbidity that lead to worse outcomes after joint replacement. Evaluation of care packages to optimize the management of obesity and multimorbidity in diabetic patients is needed to determine whether these could improve outcomes following joint replacement.

### Supplementary data

Tables 1 and 3–7 are available as supplementary data in the online version of this article, http://dx.doi.org/10.1080/17453674.2018.1473327

Study conception and design: EL, ADB, MRW, VW, AWB.

Data acquisition: ADB, VW, AWB.

Data analysis: EL.

Data interpretation: EL, ADB, MRW, VW, AWB.

Drafting of manuscript: EL, ADB, MRW, VW, AWB.

*Acta* thanks Per Kjaersgaard-Andersen and Kjell G Nilsson reviewers for help with peer review of this study.

## Supplementary Material

IORT_A_1473327_SUPP.pdf
