# Supplementary material for: Outcomes following hip and knee replacement in diabetic versus nondiabetic patients and well versus poorly controlled diabetic patients: a prospective cohort study
Source: Acta Orthop. 2018 May 14;89(4):399–405. doi: 10.1080/17453674.2018.1473327 (PMC6066772; doi:10.1080/17453674.2018.1473327)
Supplement: IORT_A_1473327_SUPP.pdf [file IORT_A_1473327_SM7772.pdf]

# Supplementary data

**Table 1. List of medications for the management of diabetes mellitus reported by research nurse at preoperative clinic assessment**

| |
|---|
| Exenatide |
| Gliclazide |
| Glucophage ® |
| Humulin ® |
| Insulatard ® |
| Metformin |
| Mixtard ® (or Human Mixtard ®) |
| Novorapid ® |

**Table 3. Outcomes by diabetic status**

| | Nondiabetic patients n = 523 | | Diabetic patients n = 64 | | Unadjusted model | Adjusted model [a] | Adjusted model [b] |
|---|---|---|---|---|---|---|---|
| | n | Summary | n | Summary | p-value | p-value | p-value |
| Inpatient period: | | | | | | | |
| Length of hospitalization (days), median (IQR) | 523 | 4 (4–6) | 64 | 5 (4–7) | 0.004 [c] | 0.02 [d] | 0.3 [d] |
| VAS Pain [e] on movement in operated joint, mean (SD) | 515 | 44 (19) | 62 | 50 (20) | 0.03 | 0.06 | 0.4 |
| VAS Pain at rest in operated joint, mean (SD) | 509 | 32 (20) | 62 | 40 (20) | 0.004 | 0.01 | 0.2 |
| At 3-month assessment: | | | | | | | |
| Infection since surgery, n (%) | 6 | (1.2) | 1 | (1.6) | 0.6 | | |
| Dislocation since surgery, n (%) | 2 | (0.4) | 0 | (0.0) | – | | |
| Deep vein thrombosis since surgery, n (%) | 1 | (0.2) | 1 | (0.2) | 0.2 | | |
| Pulmonary embolism since surgery, n (%) | 4 | (0.8) | 0 | (0.0) | 0.9 | | |
| Sciatic nerve damage since surgery, n (%) | 0 | (0.0) | 0 | (0.0) | – | | |
| Femoral nerve damage since surgery, n (%) | 0 | (0.0) | 0 | (0.0) | – | | |
| Obturator nerve damage since surgery, n (%) | 0 | (0.0) | 0 | (0.0) | – | | |
| Hospital readmission because of operated joint, n (%) | 11 | (2.1) | 1 | (1.6) | 0.8 | | |
| Further surgery on operated joint, n (%) | 10 | (1.9) | 1 | (1.6) | 0.9 | | |
| WOMAC [f] Pain, mean (SD) | 470 | 79 (20) | 57 | 76 (20) | 0.3 | | |
| WOMAC Function, mean (SD) | 438 | 77 (19) | 52 | 77 (18) | 0.9 | | |
| WOMAC Stiffness, mean (SD) | 442 | 70 (20) | 53 | 69 (24) | 0.7 | | |
| WOMAC Total, mean (SD) | 425 | 77 (18) | 51 | 76 (18) | 0.8 | | |
| At 6-month assessment: | | | | | | | |
| WOMAC Pain, mean (SD) | 469 | 83 (19) | 59 | 80 (21) | 0.2 | | |
| WOMAC Function, median (IQR) | 429 | 88 (71–96) | 52 | 82 (67–94) | 0.1 | | |
| WOMAC Stiffness, mean (SD) | 423 | 76 (21) | 51 | 73 (20) | 0.5 | | |
| WOMAC Total, mean (SD) | 416 | 82 (18) | 49 | 77 (20) | 0.08 | 0.2 | 0.9 |
| At 12-month assessment: | | | | | | | |
| Infection since surgery, n (%) | 11 | (2.1) | 1 | (1.6) | 0.8 | | |
| Dislocation since surgery, n (%) | 2 | (0.4) | 0 | (0.0) | – | | |
| Deep vein thrombosis since surgery, n (%) | 12 | (2.3) | 2 | (3.1) | 0.6 | | |
| Pulmonary embolism since surgery, n (%) | 10 | (1.9) | 1 | (1.6) | 1.0 | | |
| Sciatic nerve damage since surgery, n (%) | 5 | (1.0) | 1 | (1.6) | 0.5 | | |
| Femoral nerve damage since surgery, n (%) | 5 | (1.0) | 1 | (1.6) | 0.5 | | |
| Obturator nerve damage since surgery, n (%) | 4 | (0.8) | 1 | (1.6) | 0.4 | | |
| Hospital readmission because of operated joint, n (%) | 34 | (6.5) | 5 | (7.8) | 0.6 | | |
| Further surgery on operated joint, n (%) | 22 | (4.2) | 4 | (6.2) | 0.3 | | |
| Number of inpatient stays, median (IQR) | 493 | 0 (0–0) | 58 | 0 (0–0) | 0.2 [c] | | |
| Number of day case visits, median (IQR) | 493 | 0 (0–0) | 58 | 0 (0–0) | 0.4 [c] | | |
| Number of outpatient visits, median (IQR) | 493 | 2 (1–3) | 58 | 2 (1–3) | 0.8 [c] | | |
| WOMAC Pain, median (IQR) | 493 | 95 (75–100) | 58 | 85 (70–95) | 0.01 [c] | 0.03 [d] | 0.5 [d] |
| WOMAC Function, median (IQR) | 468 | 90 (73–97) | 55 | 79 (58–93) | 0.02 [c] | 0.08 [d] | 0.8 [d] |
| WOMAC Stiffness, median (IQR) | 467 | 88 (75–100) | 54 | 75 (50–100) | 0.02 [c] | 0.07 [d] | 0.5 [d] |
| WOMAC Total, median (IQR) | 456 | 90 (75–97) | 51 | 81 (60–93) | 0.01 [c] | 0.05 [d] | 0.7 [d] |

IQR: interquartile range
[a] Linear regression model included adjustments for age, sex, site of surgery, and trial intervention.
[b] Linear regression model included adjustments for age, sex, site of surgery, and trial intervention, BMI, and number of comorbidities (FCI).
[c] Mann–Whitney test, otherwise Student's t-test or Fisher's exact test.
[d] Ordered logistic regression model.
[e] Pain Visual Analogue Scale (0–100 best to worst).
[f] Western Ontario and McMaster Universities Osteoarthritis Index (0–100 worst to best pain, function, stiffness, or total score).

**Table 4. Multiple imputation-outcomes by diabetic status**

| | Nondiabetic patients n = 523 | Diabetic patients n = 64 | Unadjusted model p-value | Adjusted model [a] p-value | Adjusted model [b] p-value |
|---|---|---|---|---|---|
| Inpatient period: | | | | | |
| VAS Pain [e] on movement in operated joint, mean (SD) | 44 (19) | 50 (20) | 0.02 | 0.04 | 0.4 |
| VAS Pain at rest in operated joint, mean (SD) | 32 (20) | 40 (20) | 0.002 | 0.008 | 0.1 |
| At 3-month assessment: | | | | | |
| WOMAC [f] Pain, mean (SD) | 78 (20) | 75 (20) | 0.3 | | |
| WOMAC Function, mean (SD) | 76 (19) | 75 (19) | 0.7 | | |
| WOMAC Stiffness, mean (SD) | 69 (21) | 68 (24) | 0.6 | | |
| WOMAC Total, mean (SD) | 76 (18) | 75 (18) | 0.6 | | |
| At 6-month assessment: | | | | | |
| WOMAC Pain, mean (SD) | 83 (19) | 79 (21) | 0.1 | | |
| WOMAC Function, median (IQR) | 86 (68–95) | 78 (59–93) | 0.07 [c] | 0.2 [c] | 0.9 [c] |
| WOMAC Stiffness, mean (SD) | 75 (21) | 73 (21) | 0.5 | | |
| WOMAC Total, mean (SD) | 80 (18) | 75 (21) | 0.03 | 0.08 | 0.8 |
| At 12-month assessment: | | | | | |
| Number of inpatient stays, median (IQR) | 0 (0–0) | 0 (0,0) | 0.1 [d] | | |
| Number of day case visits, median (IQR) | 0 (0–0) | 0 (0,0) | 0.4 [d] | | |
| Number of outpatient visits, median (IQR) | 2 (1–3) | 2 (1–3) | 0.8 [d] | | |
| WOMAC Pain, median (IQR) | 95 (75–100) | 85 (70–96) | 0.02 [c] | 0.02 [d] | 0.5 [d] |
| WOMAC Function, median (IQR) | 89 (71–97) | 79 (58–92) | 0.02 [c] | 0.06 [d] | 0.8 [d] |
| WOMAC Stiffness, median (IQR) | 79 (75–100) | 75 (50–96) | 0.06 [c] | 0.1 [d] | 0.7 [d] |
| WOMAC Total, median (IQR) | 89 (74–97) | 80 (59–93) | 0.01 [c] | 0.04 [d] | 0.6 [d] |

[a] Linear regression model included adjustments for age, sex, site of surgery, and trial intervention.
[b] Linear regression model included adjustments for age, sex, site of surgery, and trial intervention, BMI, and number of comorbidities (FCI).
[c] Ordered logistic regression model.
[d] Poisson regression model.
[e] Pain Visual Analogue Scale (0–100 best to worst).
[f] Western Ontario and McMaster Universities Osteoarthritis Index (0–100 worst to best pain, function, stiffness or total score).

**Table 5. Preoperative characteristics of diabetics patients (n = 64) by HbA1c status**

| | HbA1c ≤ 7% n = 39 | HbA1c > 7% n = 25 |
|---|---|---|
| Trial intervention, n | | |
| Injection | 18 | 13 |
| Standard care | 21 | 12 |
| Primary total joint replacement, n | | |
| Hip | 17 | 7 |
| Knee | 22 | 18 |
| Age, mean, SD | 70 (8) | 70 (7) |
| Sex, n | | |
| Female | 17 | 13 |
| Male | 22 | 12 |
| BMI, mean, SD | 34 (6) | 34 (7) |
| ≤ 30, n | 12 | 9 |
| > 30, n | 27 | 16 |
| EQ VAS [a], n | 36 | 25 |
| mean (SD) | 62 (20) | 67 (18) |
| EQ-5D Score [b], n | 35 | 25 |
| mean, SD | 34 (0.31) | 41 (0.31) |
| FCI [c] (number of comorbidities), n | | |
| < 2 | 8 | 6 |
| 2 | 7 | 5 |
| 3 | 7 | 6 |
| > 3 | 17 | 8 |
| HADS [d] Total, n | 37 | 25 |
| mean (SD) | 13 (6) | 13 (6) |
| WOMAC [e] Pain, n | 39 | 25 |
| mean (SD) | 41 (14) | 41 (17) |
| WOMAC Function, n | 35 | 24 |
| mean (SD) | 42 (16) | 45 (18) |
| WOMAC Stiffness, n | 36 | 25 |
| mean (SD) | 42 (14) | 48 (23) |
| WOMAC Total, n | 34 | 24 |
| mean (SD) | 43 (13) | 45 (16) |
| HbA1c mmol/mol, n | 39 | 25 |
| mean (SD) | 45 (5) | 66 (10) |
| mean % (SD) | 6 (0.4) | 8.2 (1) |
| Treated for diabetes, n | | |
| None | 21 | 5 |
| Treated | 18 | 20 |
| If treated, type of management, n | | |
| Tablets | 17 | 14 |
| Tablets + insulin | 1 | 5 |
| Tablets + exenatide | 0 | 1 |

[a] EQ visual analogue scale (0–100, worst to best health state).
[b] EQ-5D 3L descriptive system.
[c] Modified Functional Co-morbidity Index without BMI and diabetes diagnoses (categorized sum of the 16 remaining diagnoses).
[d] Hospital Anxiety and Depression Scale with anxiety and depression scores combined (0–42, best to worst distress state).
[e] Western Ontario and McMaster Universities Osteoarthritis Index (0–100 worst to best pain, function, stiffness or total score).

**Table 6. Outcomes by HbA1c status (≤ 7% vs. > 7%) for diabetic patients (n = 64)**

| | HbA1c ≤ 7% n = 39 n | Summary | HbA1c > 7% n = 25 n | Summary | Unadjusted model p-value | Adjusted model [a] p-value | Adjusted model [b] p-value |
|---|---|---|---|---|---|---|---|
| Inpatient period: | | | | | | | |
| Length of hospitalization (days), median (IQR) | 39 | 5 (4–8) | 25 | 5 (4–6) | 0.3 [c] | | |
| VAS [d] Pain on movement in operated join, mean (SD) | 38 | 5 (2) | 24 | 5 (2) | 0.4 | | |
| VAS Pain at rest in operated join, mean (SD) | 38 | 4 (2) | 24 | 4 (2) | 0.5 | | |
| Postoperative HbA1c (mmol/mol), mean (SD) | 37 | 45 (7.0) | 24 | 70 (17.1) | < 0.001 | < 0.001 | < 0.001 |
| Postoperative HbA1c (%), mean (SD) | 37 | 6.2 (0.6) | 24 | 8.5 (1.6) | < 0.001 | < 0.001 | < 0.001 |
| At 3-month assessment: | | | | | | | |
| WOMAC [e] Pain, mean (SD) | 34 | 80 (18) | 23 | 69 (22) | 0.04 | 0.2 | 0.1 |
| WOMAC Function, mean (SD) | 32 | 79 (17) | 20 | 74 (20) | 0.4 | | |
| WOMAC Stiffness, mean (SD) | 32 | 73 (22) | 21 | 61 (26) | 0.07 | 0.3 | 0.2 |
| WOMAC Total, mean (SD) | 32 | 79 (17) | 20 | 73 (19) | 0.2 | | |
| At 6-month assessment: | | | | | | | |
| WOMAC Pain, median (IQR) | 35 | 90 (70–100) | 24 | 78 (60–88) | 0.01 [c] | 0.1 f | 0.03 [f] |
| WOMAC Function, median (IQR) | 30 | 88 (72–94) | 22 | 77 (47–83) | 0.1 [c] | | |
| WOMAC Stiffness, mean (SD) | 29 | 78 (18) | 22 | 68 (21) | 0.07 | 0.3 | 0.05 |
| WOMAC Total, median (IQR)) | 27 | 89 (71–94) | 22 | 78 (53–86) | 0.1 [c] | | |
| At 12-month assessment: | | | | | | | |
| Number of complications, median (IQR) | 34 | 0 (0–0) | 24 | 0 (0–0) | 0.9 [c] | | |
| Number of inpatient stays, median (IQR) | 34 | 0 (0–0) | 24 | 0 (0–0) | 0.8 [c] | | |
| Number of day case visits, median (IQR) | 34 | 0 (0–0) | 24 | 0 (0–0) | 0.4 [c] | | |
| Number of outpatient visits, median (IQR) | 34 | 2 (1–3) | 24 | 2 (1–3) | 0.8 [c] | | |
| WOMAC Pain, median (IQR) | 34 | 90 (70–100) | 24 | 80 (58–95) | 0.1 [c] | | |
| WOMAC Function, median (IQR) | 33 | 81 (58–94) | 22 | 78 (58–91) | 0.6 [c] | | |
| WOMAC Stiffness, mean (SD) | 33 | 78 (21) | 21 | 61 (21) | 0.005 | 0.01 | 0.004 |
| WOMAC Total, median (IQR)h) | 32 | 83 (63–93) | 20 | 74 (55–83) | 0.2 [c] | | |

[a] Linear regression model included adjustments for age, sex, site of surgery, and trial intervention.
[b] Linear regression model included adjustments for age, sex, site of surgery, and trial intervention, BMI, and number of comorbidities (FCI).
[c] Mann–Whitney test, otherwise Student's t-test.
[d] Pain Visual Analogue Scale (0–100 best to worst).
[e] Western Ontario and McMaster Universities Osteoarthritis Index (0–100 worst to best pain, function, stiffness, or total score).
[f] Ordered logistic regression model.

**Table 7. Multiple imputation-outcomes by HbA1c status (≤ 7% vs. > 7%) for diabetic patients (n = 64)**

| | HbA1c ≤ 7% n = 39 | HbA1c > 7% n = 25 | Unadjusted model p-value | Adjusted model [a] p-value | Adjusted model [b] p-value |
|---|---|---|---|---|---|
| Inpatient period: | | | | | |
| VAS [e] Pain on movement in operated join, mean (SD) | 5 (2) | 5 (2) | 0.5 | | |
| VAS Pain at rest in operated join, mean (SD) | 4 (2) | 4 (2) | 0.7 | | |
| Postoperative HbA1c (mmol/mol), mean (SD) | 45 (7) | 69 (17) | < 0.001 | < 0.001 | < 0.001 |
| Postoperative HbA1c (%), mean (SD) | 6.2 (0.7) | 8.5 (1.6) | < 0.001 | < 0.001 | < 0.001 |
| At 3-month assessment: | | | | | |
| WOMAC [f] Pain, mean (SD) | 80 (17) | 69 (22) | 0.05 | 0.1 | 0.08 |
| WOMAC Function, mean (SD) | 77 (17) | 72 (21) | 0.3 | | |
| WOMAC Stiffness, mean (SD) | 71 (22) | 62 (26) | 0.2 | | |
| WOMAC Total, mean (SD) | 77 (17) | 71 (20) | 0.2 | | |
| At 6-month assessment: | | | | | |
| WOMAC Pain, median (IQR) | 90 (69–100) | 80 (65–88) | 0.07 [c] | 0.1 [c] | 0.05 [c] |
| WOMAC Function, median (IQR) | 82 (61–94) | 76 (47–83) | 0.5 [c] | | |
| WOMAC Stiffness, mean (SD) | 76 (20) | 67 (22) | 0.1 | | |
| WOMAC Total, median (IQR) | 85 (65–94) | 76 (53–85) | 0.3 [c] | | |
| At 12-month assessment: | | | | | |
| Number of inpatient stays, median (IQR) | 0 (0–0) | 0 (0–0) | 0.8 [d] | | |
| Number of day case visits, median (IQR) | 0 (0–0) | 0 (0–0) | 1.0 [d] | | |
| Number of outpatient visits, median (IQR) | 2 (1–3) | 2 (1–3) | 1.0 [d] | | |
| WOMAC Pain, median (IQR) | 88 (70–100) | 80 (60–95) | 0.2 [c] | | |
| WOMAC Function, median (IQR) | 81 (59–94) | 76 (55–88) | 0.5 [c] | | |
| WOMAC Stiffness, mean (SD) | 77 (22) | 65 (22) | 0.03 | 0.04 | 0.02 |
| WOMAC Total, median (IQR) | 85 (61–93) | 75 (56, 86) | 0.1 [c] | | |

[a] Linear regression model included adjustments for age, sex, site of surgery, and trial intervention.
[b] Linear regression model included adjustments for age, sex, site of surgery, and trial intervention, BMI, and number of comorbidities (FCI).
[c] Ordered logistic regression model.
[d] Poisson regression model.
[e] Pain Visual Analogue Scale (0–100 best to worst).
[f] Western Ontario and McMaster Universities Osteoarthritis Index (0–100 worst to best pain, function, stiffness or total score).
